# Sulcal pits as potential markers of early sex-related human brain differences in healthy adults

**DOI:** 10.1186/s13293-025-00733-4

**Published:** 2025-07-22

**Authors:** Noemí Hostalet, Pilar Salgado-Pineda, Yasser Alemán-Gómez, Lluís Cobos-Aumatell, Alejandro Sotero-Moreno, Irene París-Gómez, Ana Aquino-Servín, Erick J. Canales-Rodríguez, Amalia Guerrero-Pedraza, Salvador Sarró, Jordi Ortiz-Gil, Kiho Im, Neus Martínez-Abadías, Edith Pomarol-Clotet, Mar Fatjó-Vilas

**Affiliations:** 1 FIDMAG Germanes Hospitalàries Research Foundation, Barcelona, Spain; 2 Departament de Biologia Evolutiva, Ecologia i Ciències Ambientals (BEECA), Facultat de Biologia, Universitat de Barcelona (UB), Barcelona, Spain; 3 Centro de Investigación Biomédica en Red de Salud Mental (CIBERSAM), Instituto de Salud Carlos III, Madrid, Spain; 4 Programa de Doctorat en Biomedicina, Universitat de Barcelona (UB), Barcelona, Spain; 5 Connectomics Lab, Department of Radiology, Centre Hospitalier Universitaire Vaudois (CHUV) and University of Lausanne (UNIL), Lausanne, Switzerland; 6 Freelance computing assessor, Barcelona, Spain; 7 Signal Processing Laboratory (LTS5), École Polytechnique Fédérale de Lausanne (EPFL), Lausanne, Switzerland; 8 Fundación Hospitalarias Sant Boi, Sant Boi de Llobregat, Barcelona, Spain; 9 GRAD Atenció a la Diversitat, Psychology Department, Faculty of Education, Translation, Sports and Psychology, Universitat de Vic - Universitat Central de Catalunya, Vic, Spain; 10 Fetal-Neonatal Neuroimaging and Developmental Science Center, Boston Children’s Hospital, Harvard Medical School, Boston, USA; 11 Division of Newborn Medicine, Department of Pediatrics, Boston Children’s Hospital, Harvard Medical School, Boston, USA; 12 Department of Radiology, Centre Hospitalier Universitaire Vaudois (CHUV) and University of Lausanne (UNIL), Lausanne, Switzerland; 13 Computational Medical Imaging and Machine Learning Section, Center for Biomedical Imaging (CIBM), Lausanne, Switzerland

## Background

Differences in brain structure between sexes are present across developmental stages throughout the lifespan and may stem from distinct neurodevelopmental trajectories in males and females [1–3]. These pathways are shaped by genetic and environmental factors, to which males and females may experience different levels of exposure and respond differentially [4, 5]. Such developmental scenarios may contribute to the neuroanatomical sex-based differences in the general population, but also to the patterns of prevalence and symptoms in neurodevelopmental disorders [6].

Among the sex differences in brain structures detected in adults, a meta-analysis revealed that males generally exhibit higher overall grey matter and white matter volumes compared to females at the whole-brain level [7]. At a regional level, these differences were observed in the cortex (including the insular, cingulate, and occipital regions) as well as in subcortical structures (such as the amygdala, thalamus, hippocampus, and putamen) [7].

To gain a deeper understanding of these sex differences in adulthood, it is crucial to examine earlier stages of neurodevelopment as differences in brain structure between sexes have also been reported in adolescence [8, 9], childhood [10, 11], and at prenatal stages [12]. This supports the notion that the biological mechanisms driving these differences likely begin to operate during prenatal development and persist throughout the lifespan [1–3].

More concretely, sex differences in brain development during the prenatal period are influenced by the interplay of different factors acting at critical developmental windows [4, 5]. For instance, around the 8th gestational week (GW), the differentiation of testes in male fetuses leads to a peak in testosterone levels between 14th − 18th GW which, together with other sex steroids differentially expressed in males and females during this period, plays a crucial role in brain sex differentiation [13]. Coinciding with this critical window of hormone activity, structural brain differences become evident by the 18th GW, laying the foundation for sex-specific developmental patterns that persist from mid-gestation onwards [14].

Despite the available data, significant gaps remain in our understanding of the origins of sex-based neurodevelopmental trajectories. One key challenge lies in improving our understanding of how early brain developmental mechanisms contribute to the variability observed in the adult brain.

During the fetal period, the human brain cortex transitions from a lissencephalic to a folded structure composed of gyri and sulci, and the sulcal pits are considered indicative of the initial folding points of the sulci [15]. The sulcation process of the cortex follows a specific temporary sequence [16, 17]. It begins at the 9th GW with the formation of the primary sulci, the most stable sulci across individuals [18–22], followed by the secondary and tertiary sulci emerging at later stages of prenatal and postnatal neurodevelopment [21, 23]. Kruggel et al. [24] illustrated that primary sulci are situated deeper within the cortex than the subsequent emerging sulci. This suggests a hierarchical organization in sulci formation, where primary sulci form the deepest folds followed by progressively shallower secondary and tertiary sulci; indicating the possibility of establishing a sequential sulci formation based on their depth.

Some sex differences have been reported regarding the temporal emergence of the sulci. Specifically, female fetuses exhibited a tendency for an earlier emergence of sulci in the right superior temporal sulcus, a higher temporal variability in the right precentral sulcus and a lower temporal variability in the right intraparietal sulcus when compared to male fetuses [20]. In adults, sex differences have been described in the width and length of specific sulci, such as the inferiorprecentral or the lateral sulci [25, 26] and one study achieved a high accuracy rate (85%) in sex classification based on the shape of sulci at whole-brain level in adults [27]. However, these differences shown on the sulci may be affected by age, since a sex-dependent effect of aging on sulci morphometry has been reported [28]. Therefore, given the variability of sulcal morphology, there is a growing interest in identifying more stable anatomical markers closely linked to early brain development. In this regard, the sulcal pits, defined as the deepest points within the cortical sulci, have emerged as potential candidates. The sulcal pits correspond to the positions of maximum depth of sulcal basins, they are thought to be genetically determined and exhibit consistent spatial patterns from birth through adulthood [29–31]. Their stability and early emergence suggest that sulcal pits could serve as reliable indicators of neurodevelopmental processes, potentially aiding the detection of deviations associated with developmental disorders [32, 33].

To date, although several studies have analyzed the patterns of sulcal pits in various neurodevelopmental disorders such as autism, attention deficit disorder, bipolar disorder or dyslexia [34–38], none of them has accounted for the effect of sex despite the growing evidence of brain sex differences throughout lifespan [1]. Only one methodological study, which focused on developing a new technique for analyzing the patterns of sulcal pits in a sample of healthy individuals [39], examined differences between males and females, revealing distinct patterns between sexes in the left collateral sulcus and the right lateral occipital, cingulate, and middle frontal sulci. In addition, understanding the biological mechanisms leading to differences in the patterns of sulcal pits is limited because the relationship between the distribution and depth of sulcal pits and other neuroanatomical measures, remains unexplored.

Based on all the above-described, the general objectives of this study were, first, to examine sex-related variations in the patterns of sulcal pits in a sample of healthy individuals and assess their association with other neuroanatomical features; and second, to investigate the temporal emergence of these differences and their potential functional implications. Acquiring this knowledge could enhance the interpretation of existing data on brain development and neurodevelopmental disorders, thereby informing and improving translational research strategies.

In pursuit of the first objective, the specific aims were to: (i) examine sex differences in the frequency of sulcal pits at the hemispheric level; and (ii) determine whether these global distribution patterns correlate with other neuroanatomical measures, such as cortical thickness and cortical surface area.

For the second objective, the specific aims were to: (iii) explore the potential temporal window during which sex differences in sulcal pits patterns may have emerged; and (iv) analyze the potential functional implications of these differences. To address these last two specific aims, the brain was parcellated into distinct regions (areals), which were then categorized into three depth-based clusters: deep, intermediate, and shallow. Depth-based classification allowed us to examine the relationship between cortical depth and temporal emergence, as proposed in previous studies [24, 36]. These studies suggested that the earliest-developing brain regions corresponded to the deepest clusters, followed sequentially by the intermediate and shallow clusters. Accordingly, we examined differences in both the frequency of sulcal pits within each depth cluster and the variation in sulcal pits depth across areals. Finally, each areal was mapped to Destrieux regions and Brodmann areas to discuss further the potential anatomical and functional correlates of the observed sex differences in sulcal pits depth.

Based on the studies mentioned above that show sex differences in prenatal and adult sulci patterns [25–27], and the study that identified differences in the patterns of sulcal pits between adult males and females [39], we hypothesized that our approach would detect sex differences in the frequency and depth of sulcal pits between males and females. These differences may reflect time-specific neurodevelopmental processes and could also be linked to adult variability in cortical surface area and thickness.

## Methods

### Sample

The sample comprised a total of 190 healthy individuals, 93 males and 97 females, recruited from FIDMAG Hermanas Hospitalarias Research Foundation through public advertising in the province of Barcelona. All participants met the following inclusion criteria: not having a current or antecedent history or first-degree family history of psychiatric disorders; European ancestry; age between 18 and 65 years old; right-handedness; and estimated intelligence quotient (IQ) > 70, assessed with the Test de Acentuación de Palabras (TAP) [40], an adapted Spanish version of the National Adult Reading Test (NART) [41]. Exclusion criteria included drug or alcohol abuse and a history of neurological damage. All participants provided written consent after being informed about the study procedures and implications.

The sample characteristics are provided in Table 1. No significant differences between males and females regarding age (Student’s t-test, t = -1.265; *p* = 0.2) and TAP (Student’s t-test, t = 0.02; *p* = 0.99) were found.

**Table 1:** Description of the individuals included in the sample of the study

	Males (*n* = 93)	Females (*n* = 97)
Mean age (SD) [age range]	36.62 (10.7) [18.59–63.74]	38.69 (11.8) [19.14–64.77]
Mean TAP (SD) [TAP range]	23.49^*^ (4.16) [12–30]	23.49^*^ (3.76) [9–30]

^*^A TAP score of 23 corresponds to an estimated Intelligence Quotient (IQ) of 102

### MRI acquisition

All participants underwent a neuroimaging protocol in a 1.5 Tesla GE Sigma scanner at Hospital Sant Joan de Déu (Esplugues de Llobregat, Spain). High-resolution T1 weighted (T1w) head magnetic-resonance images (MRI) were obtained with the following acquisition parameters: matrix size 512 × 512; 180 contiguous axial slices; voxel resolution 0.47 × 0.47 × 1 mm3; echo (TE), repetition (TR) and inversion (TI) times, (TE/TR/TI) = 3.93ms/2000ms/710ms, respectively; and flip angle 15º.

### MRI processing

T1w MRIs were processed through the recon-all pipeline of FreeSurfer (v. 5.3), which included motion correction, removal of non-brain tissue, automated Talairach transformation, tessellation of the grey and white matter boundaries and topological correction to rectify holes, intersections and isolated vertices. The triangulated mesh representing the cortical surface was achieved using a deformation algorithm, and the mean surface area and mean cortical thickness were obtained for all subjects. All the images included in this study complied with the standardized quality control protocols from the ENIGMA consortium (http://enigma.ini.usc.edu/protocols/imaging-protocols).

### Sulcal pits extraction

The sulcal pits were extracted from the mesh of the white matter surface for each hemisphere, using the process “Sulcal pits extraction” implemented in BrainVisa 4.5 (https://brainvisa.info/web/). The process is detailed in Auzias et al. [31].

Briefly, a depth map was first generated by using the Depth Potential Function (DPF) [42]. Then, a watershed algorithm divided large sulci into sulcal basins based on ridge height (R), basin area (A), and the geodesic distance between two pits (D). The parameters of group Fiedler length (gFL) and group average surface area (gSA) were calculated based on our sample values to optimize the process, as suggested in Auzias et al. [31], obtaining averaged values between hemispheres of gFL = 219.84 mm and gSA = 81625.11 mm^2^. On the other hand, the default thresholds of ridge height (ThrR), area (ThrA) and distance (ThrD) for basin merging implemented in BrainVisa (ThR = 1.5 mm, ThD = 20 mm, ThA = 50 mm^2^) were maintained due to their proven good performance for sulcal pits extraction [31]. The use of DPF for the extraction of sulcal pits ensures that the procedure is independent of brain size, as it does not affect the computation of depth in this method [31]. Furthermore, to ensure that noisy pits were not extracted on the bumps of shallower sulci, a restriction to consider exclusively sulcal pits with positive DPF values was applied.

### Group areals definition

**Fig. 1:**
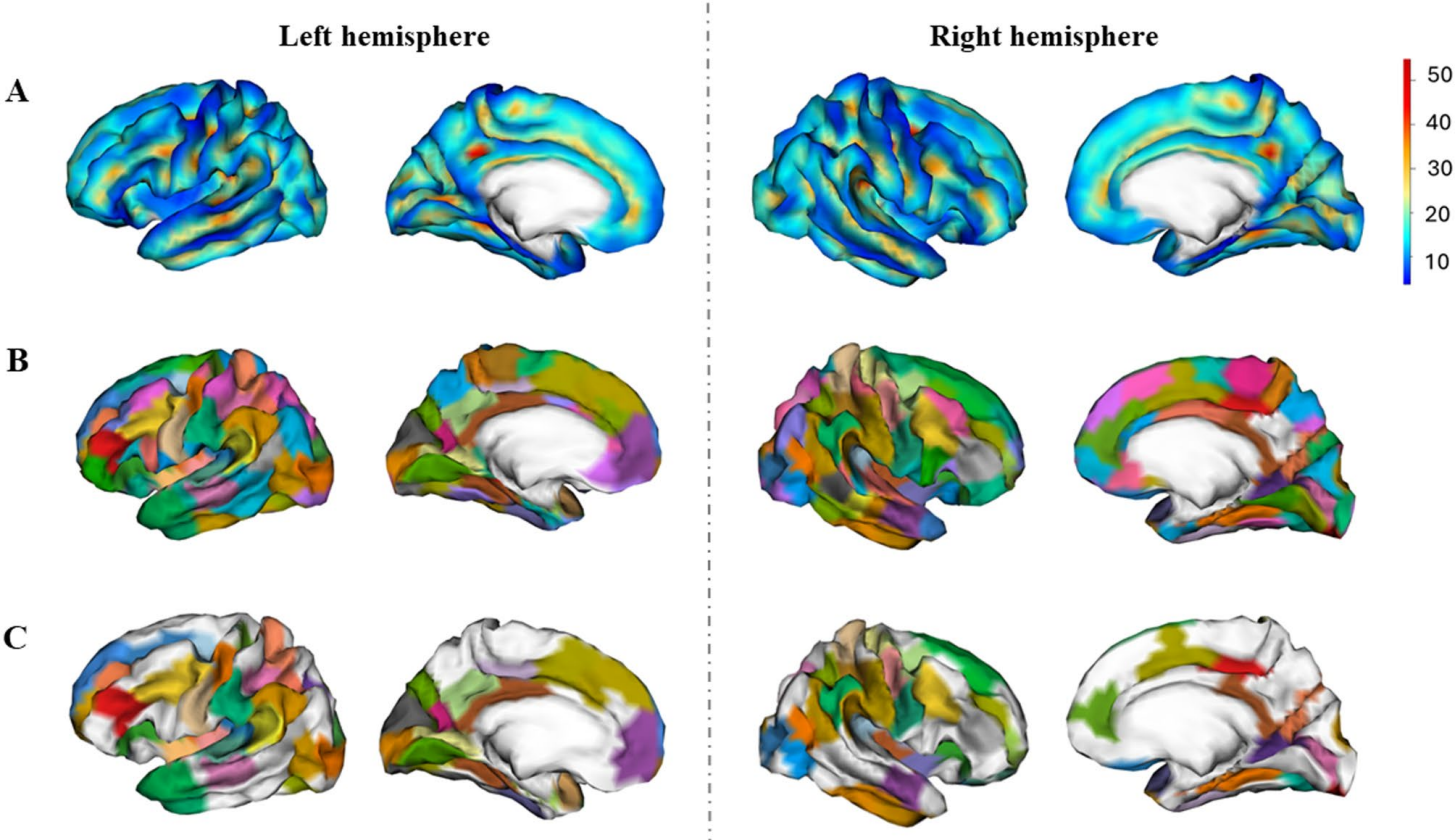
Density and areals maps. After individual extraction of sulcal pits, the individual maps were smoothed and summed to obtain a density map, showing the probability of sulcal pits occurrence in the whole sample (**A**). Then, a watershed algorithm was applied to obtain the map of the areals (**B**). Finally, those areals in which at least 80% of the sample presented at least one sulcal pit were selected for further analyses (**C**)

To compare the differences in sulcal pits between sexes, we applied the method depicted in [31]. An areal map, consisting in the parcellation of the brain in smaller regions, was built considering all individuals, both males and females, so that each group equally contributed to the final parcellation. First, the sulcal pits maps were smoothed using a full-width half-maximum (FWHM) of 5 mm, maintaining the peak value at one, by using the BrainVisa process “Pits Texture Smoothing”. Second, the smoothed pits’ maps of both hemispheres were projected to the corresponding hemisphere of the Freesurfer fsaverage4 template. Third, all the individual sulcal pits maps were summed to obtain a group density map, showing the probability of sulcal pits at each vertex (Fig. 1A). Fourth, the BrainVisa process “Group Watershed” with the default parameters (ThR = 2 mm, ThD = 15 mm and ThA = 100 mm^2^) was applied to the density map to obtain the group areals (Fig. 1B). In all, 98 areals were mapped in the left hemisphere and 101 in the right.

Finally, the individual sulcal pits maps were projected into the parcellated template. As expected, due to methodological constraints, although most individuals presented one sulcal pit in each areal, some did not present any or more than one [31]. Therefore, only those areals with at least a frequency of one sulcal pit in 80% of the sample were retained for further analysis (49 for the left hemisphere and 47 for the right) (Fig. 1C). As established in previous studies, this criterion ensures that the analyses of the differences in sulcal pits are performed in the most reproducible sulci across individuals, the primary sulci, which are the first sulci to emerge [21, 32, 43].

Subsequently, to provide biological meaning to the sulcal pits on those areals, we used the Destrieux atlas [44] to describe the anatomy of the cortical regions comprised by each areal. In addition, the Brodmann atlas, as implemented in MRIcro [45], was used to determine the Brodmann area (BA) correspondence of the areals. In Supplementary Materials 1, Tables S1 – S2, a detailed description of the fsaverage4 coordinates (MNI305) comprised in each areal and their correspondence to Destrieux atlas and BA is provided for the left and right hemispheres.

### Classification of areals by their developmental emergence: depth-based clustering

To perform a refined analysis of the differences in sulcal pits related to the different stages of prenatal brain development, we classified areals into three groups based on their depth, applying a k-means algorithm as presented in Li et al. [36]. With this methodology the areals are aligned accordingly with the temporal emergence of the sulci within them. In accordance with previous studies, the deepest areals are presumed to contain the earliest-emerging sulci, while the shallowest comprise the later-emerging ones [24, 36].

K-means was performed considering the maximum depth values of the sulcal pits of all individuals in each areal, and not the average DPF value of the areal as in Li et al. [36], ensuring that the captured depth value corresponded to the fundi of the cortex and was not distorted by values of other vertices present on gyri. If an individual did not present a sulcal pit in a determined areal (7.18% of cases in the left hemisphere and 7.67% in the right hemisphere), this value was filled with the group-average DPF of the sulcal pits in that areal. When an individual presented more than one, the value of the deepest pit was considered (27.19% of cases in the left hemisphere and 26.91% in the right hemisphere). K-means was conducted with three classes (k = 3) and 100 iterations were performed. As a result, we obtained the classification of each areal in one of the 3 depth clusters (deep, intermediate and shallow) for each hemisphere. The results of the k-means are provided in Supplementary Materials 2, Figure S1 and Table S1 correspond to the left hemisphere and Figures S2 – S3 and Tables S2 – S3 to the right hemisphere. Greater values of DPF represent greater depths. Therefore, areals classified in the deepest cluster would exhibit higher DPF values compared to those classified in the intermediate and shallow clusters. For clarity, henceforth, DPF will be referred to as depth.

**Fig. 2:**
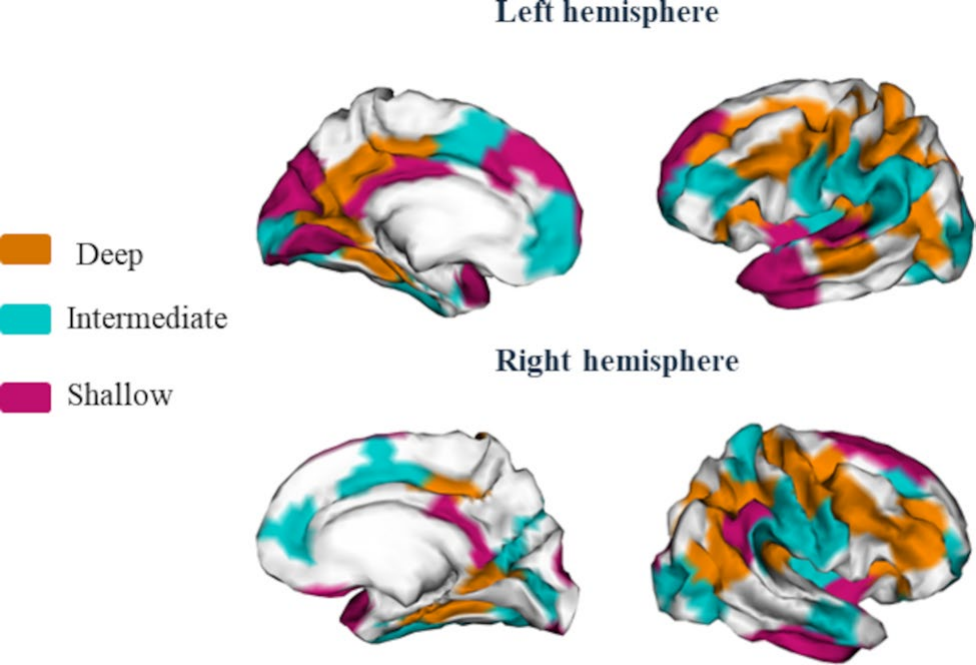
Representation of the areals in each depth cluster for the left and right hemispheres. Areals classified in the deep cluster are represented in orange, those in the intermediate cluster in blue and the shallowest areals are in pink. The medial and outer parts of the cortex are displayed (left and right parts of the figure, respectively). Non-colored regions contain areals where fewer than 80% of individuals had a sulcal pit

For the left hemisphere, the deep cluster comprised 20 areals, the intermediate 18 and the shallow 11. In the case of the right hemisphere, the deep cluster comprised 17 areals, the intermediate 20 and the shallow 9. In Fig. 2, a visualization of the areals comprised in each cluster is presented.

Supplementary Materials 1 provides a table of the classification of the areals and their mean depth for the left and right hemispheres (Table S5 and Table S6, respectively).

### Statistical analyses

To address our first objective, we examined sex differences in the frequency of sulcal pits at the hemispheric level.

First, we conducted linear regression analyses with the total number of sulcal pits per hemisphere as the dependent variable. In a first model, we included sex as the main factor, and, as covariates, age, estimated IQ, and estimated intracranial volume (ICV, defined as the total volume in the cranial cavity). Despite the stability of sulcal pits across the lifespan, we included age as a covariate due to its influence on overall variability in brain anatomy [46–48] and the potential indirect effects it may have on sulcal pits measurement. The inclusion of the estimated IQ was based on previous findings suggesting its influence on sulcal pits frequency [49]. Although pit depth may be relatively unaffected by brain size [31], sulcal pits frequency could vary with ICV. Moreover, considering the well-established sex differences in brain size [50], adjusting for ICV was essential. In a second model, we extended the analysis to explore the interaction between sex and age (sex x age), while retaining the same covariates. This allowed us to assess whether the relationship between sex and sulcal pits frequency varied across age at brain scanning.

Second, we analyzed the correlation between sulcal pits and additional neuroanatomical cortical metrics at the hemispheric level by first adjusting the mean surface area and mean cortical thickness values for ICV to mitigate potential distortions in the analyses stemming from differences in brain size between groups. To do so, a linear regression model was employed, considering mean surface area or mean cortical thickness as the dependent variables and ICV as the independent variable. The residuals of that model were then retained for subsequent analyses. Next, we used a regression model to test the correlation between the number of sulcal pits at the hemispheric level and the surface area within the corresponding hemisphere. This analysis was performed in the sex-pooled sample and stratifying the sample by sex. The same procedure was applied in the case of mean cortical thickness. Age and estimated IQ were considered as covariates and a second model to explore the interaction between age and sex was also built.

A post-hoc power analysis indicated that, with our sample size, we had 80% power to detect significant effects of sex on the number of sulcal pits for a ß of at least ± 1.629.

Regarding our second objective, we explored the potential temporal window during which differences in the frequency of sulcal pits may have emerged. Therefore, we calculated the total number of pits in the areals conforming the depth, intermediate and shallow clusters. Then, a linear model was built considering the total number of pits in each depth cluster as a dependent variable, sex as the main factor and age, estimated IQ, and ICV as covariates. A second linear model, including the interaction between age and sex, was also performed.

Subsequently, we analyzed sex differences in sulcal pits depth for each areal to topographically identify the anatomical and functional regions where these differences may be localized. First, the interquartile range (IQR) method was applied to detect potential outlier depth values in each areal and those individuals whose depth values were above or below 1.5*IQR (~ 2.7 standard deviations) were excluded. In Supplementary Materials 1, Tables S15 – S16, the percentage of outliers in each areal is presented. Next, a linear model was built considering sulcal pit depth in each areal as the dependent variable, sex as the main factor and age and estimated IQ as covariates.

For all models, we report both the raw *p*-value (p) and the multiple comparisons adjusted *p*-value (p_FDR_) obtained with the False Discovery Rate (FDR) method [51]. Statistical significance was established at p_FDR_ < 0.05. The statistical analyses were performed using R (v. 4.3.2) [52] and the results on the brain templates were plotted using *fsbrain* R package [53].

## Results

### Sex differences in the distribution of sulcal pits and their relationship with cortical measures

**Fig. 3:**
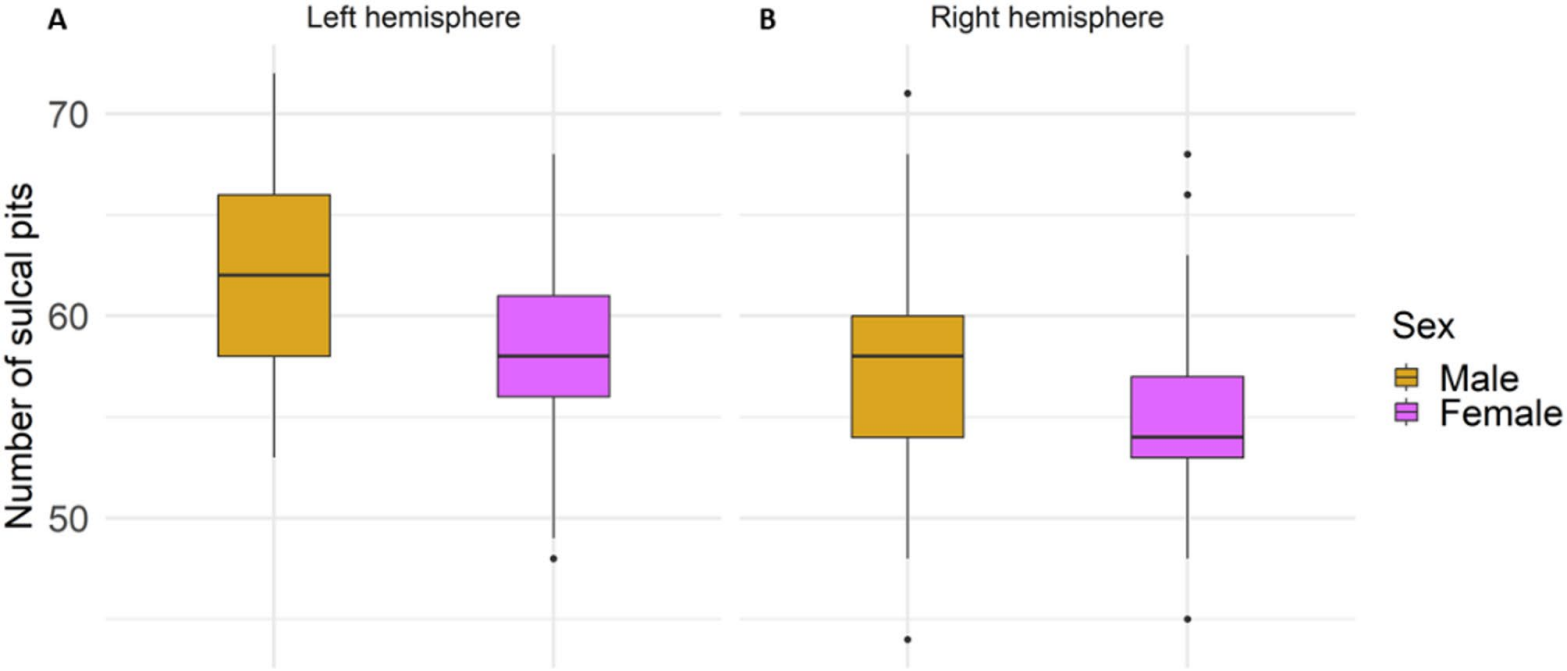
Boxplots of the number of sulcal pits in each sex for the left (**A**) and right (**B**) hemisphere

First, we analyzed the frequency of sulcal pits in each hemisphere and compared the distribution between sexes. In both hemispheres, the number of pits was higher in males (left hemisphere: mean (SD) = 62.2 (4.62); right hemisphere: mean (SD) = 57.5 (4.45)) than in females (left hemisphere: mean (SD) = 58.4 (4.06); right hemisphere: mean (SD) = 55.2 (3.94)), as shown in Fig. 3. In the left hemisphere, these differences were marginally significant after adjusting for covariates and correcting by FDR (ß = -1.81; s.e. = 0.71; *p* = 0.012; p_FDR_ = 0.06; R^2^ = 0.25), while no interaction between sex and age was found (Table S7, Supplementary Materials 1). Regarding the right hemisphere, sex did not show an effect on the number of sulcal pits (ß = 0.71; s.e. = 0.70; *p* = 0.311; p_FDR_ = 0.615), but a marginally significant interaction of sex and age emerged (ß = -0.14; s.e. = − 0.05; *p* = 0.008; p_FDR_ = 0.053; R^2^ = 0.16) (Table S8, Supplementary Materials 1). Furthermore, a significant effect of ICV on the number of sulcal pits was found in both hemispheres (Tables S7 – S8, Supplementary Materials 1).

**Fig. 4:**
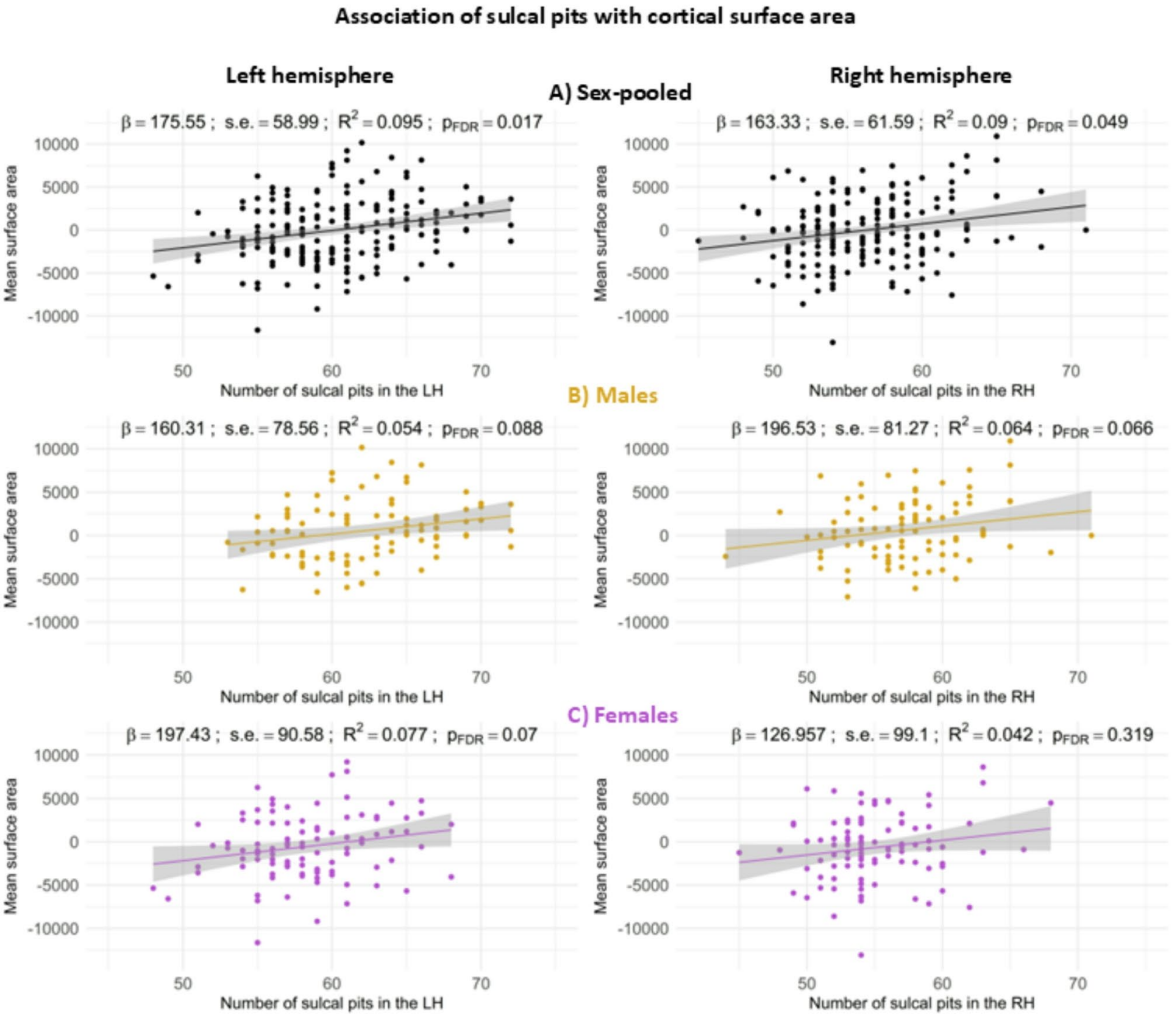
Scatterplots of the association of the number of sulcal pits in the left hemisphere (left column) and right hemisphere (right column) with mean surface area adjusted for ICV in the sex-pooled sample (**A**), males (**B**) and females (**C**)

**Fig. 5:**
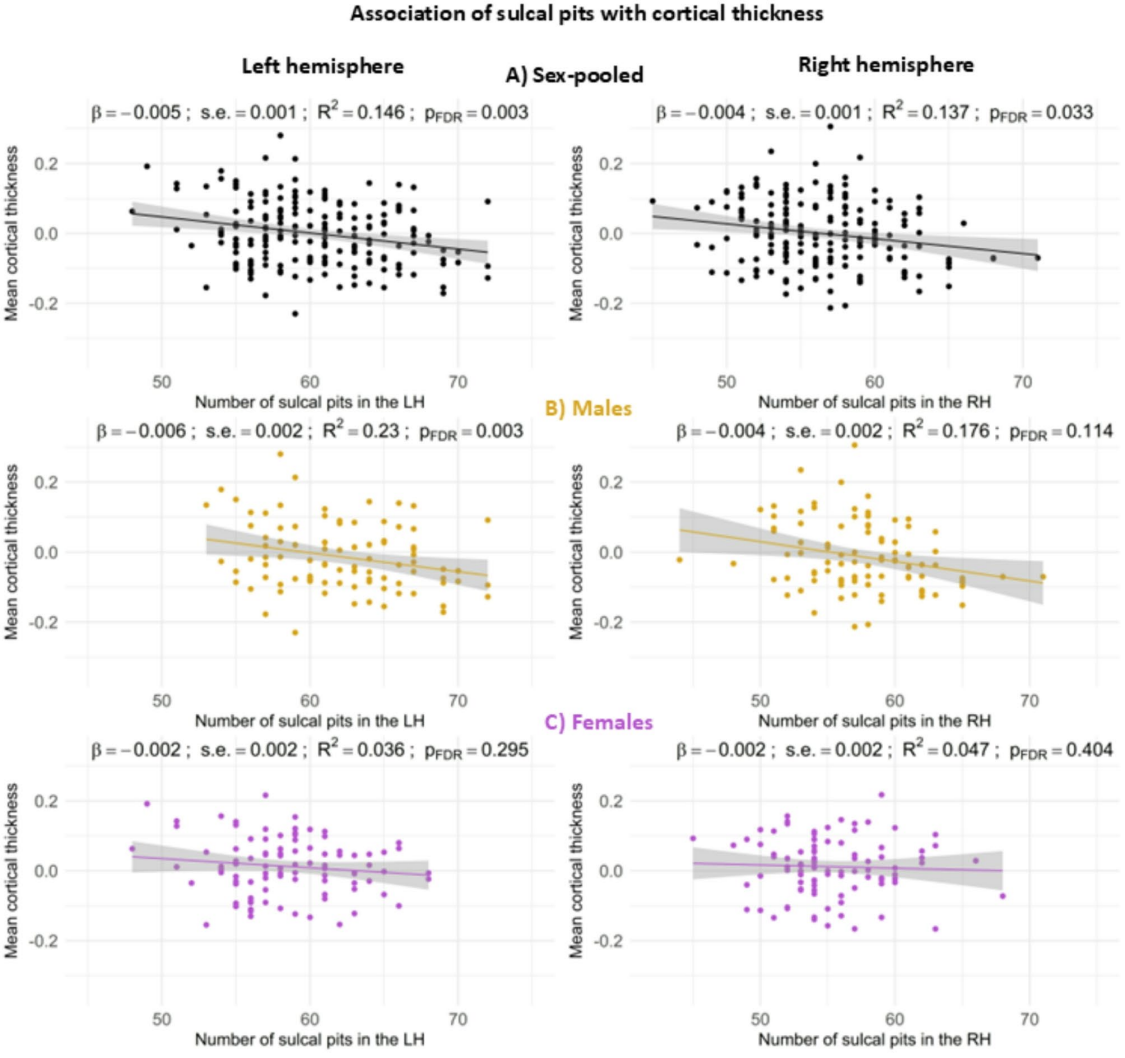
Scatterplots of the association of the number of sulcal pits in the left hemisphere (left column) and right hemisphere (right column) with mean cortical thickness adjusted for ICV in the sex-pooled sample (**A**), males (**B**) and females (**C**)

Second, we examined the association of the number of sulcal pits in each hemisphere with other cortical neuroanatomical metrics (Figs. 4 and 5). A significant effect of surface area and cortical thickness was observed on the number of sulcal pits.

As regards surface area, a greater number of pits was associated with higher mean area values in both hemispheres for the sex-pooled sample (LH *p* = 0.003; p_FDR_ = 0.017; RH *p* = 0.009; p_FDR_ = 0.049; Fig. 4A). In the left hemisphere, a marginally significant association was observed for males (*p* = 0.044; p_FDR_ = 0.08; Fig. 4B) and females (*p* = 0.032; p_FDR_ = 0.07; Fig. 4C). In the right hemisphere, a marginally significant association was observed in males (*p* = 0.018; p_FDR_ = 0.06; Fig. 4C) but not in females (Fig. 4C).

In relation to cortical thickness, a higher number of sulcal pits was associated with reduced values in both hemispheres in the sex-pooled sample (LH *p* = 0.001; p_FDR_ = 0.003; RH *p* = 0.012; p_FDR_ = 0.03; Fig. 5A), an effect likely driven by males in the left hemisphere (*p* = 0.001; p_FDR_ = 0.003; Fig. 5B), as females did not show significant associations (Fig. 5C).

Regarding the covariates, a significant effect of age on surface area and cortical thickness was found, while an interaction between age and sex was exclusively observed for cortical thickness (Tables S9 – S12, Supplementary Materials 1).

### Temporal emergence of sex differences on the distribution of sulcal pits and their potential functional implications

**Fig. 6:**
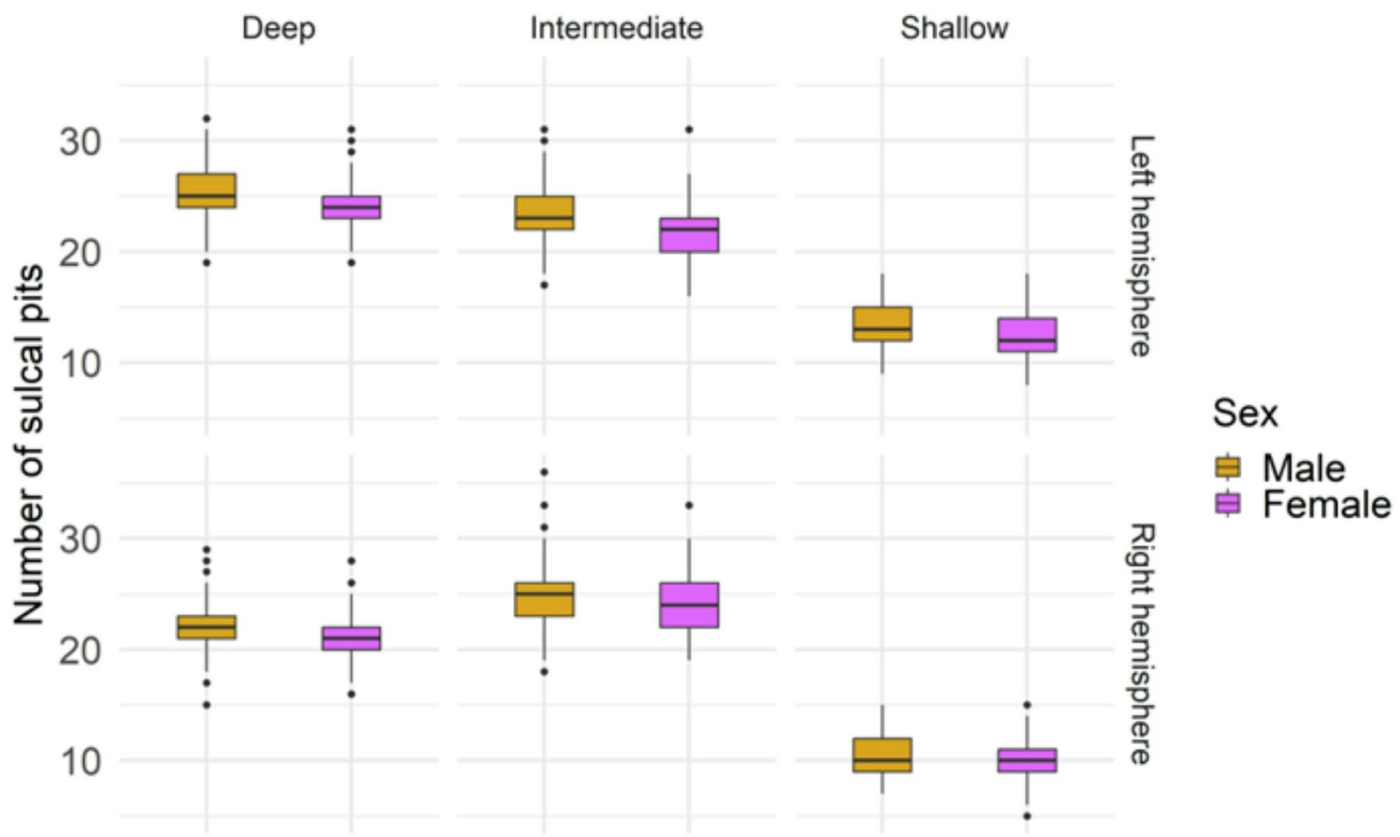
Boxplots illustrate the number of sulcal pits for males and females within the deep, intermediate, and shallow clusters for both, the left (top panel) and right (bottom panel) hemispheres

First, we examined the sex differences in the frequency of sulcal pits within the three depth clusters. Males showed a higher number of pits across all depth clusters in both hemispheres in comparison to females (see Fig. 6; Table 2). However, when adjusting for age, estimated IQ and ICV, the sex differences were no longer significant in any depth cluster, and a significant effect of ICV was found (Supplementary Materials 1, Table S7 – S8).

**Table 2:** Summary of linear regressions considering sex as a main factor and estimated IQ as a covariate. The results for sex in each cluster and hemisphere are shown. The mean number of pits and SD for each sex are also reported

		β	s.e.	*R* ^2^	*p*_FDR_	male mean (SD)	female mean (SD)
Left hemisphere	Deep	-1.15	0.36	0.041	0.004	25.3 (2.68)	24.2 (2.32)
	Intermediate	-1.40	0.38	0.057	0.0009	23.4 (2.69)	22.0 (2.56)
	Shallow	-1.24	0.29	0.080	1.8 × 10^− 4^	13.5 (2.05)	12.3 (1.93)
Right hemisphere	Deep	-0.91	0.34	0.027	0.011	22.0 (2.56)	21.1 (2.08)
	Intermediate	-0.84	0.41	0.018	0.044	24.8 (3.20)	24.0 (2.46)
	Shallow	-0.58	0.26	0.017	0.03	10.6 (1.74)	10.0 (1.79)

Next, to enhance specificity in our approach, we conducted a detailed analysis of the sulcal pits depth patterns across cortical areals. With this aim, we assessed which areals had sex-related differences in sulcal pit depth and explored their correspondence to Destrieux regions [44]. Additionally, we also provide the corresponding Brodmann areas (BA) according to the template implemented in MRIcro [45].

**Fig. 7:**
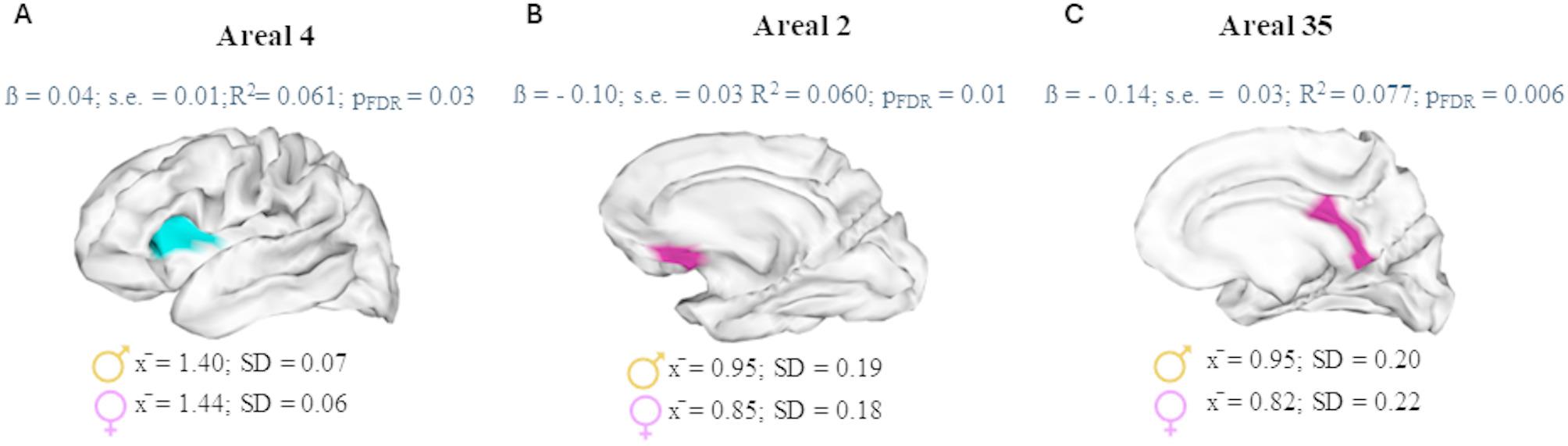
Representation of areals in which sex had a significant effect on sulcal pits depth in the left (**A**) and right hemispheres (**B **and **C**). Areal 4 comprised the horizontal ramus of the lateral sulcus and the anterior and posterior parts of the insula; areal 2, the medial orbital sulcus; and areal 35, the pericallosal sulcus and the middle and posterior parts of the cingulate sulcus. For each areal represented, the top section displays the outcomes of the linear regression model concerning the sex effect on sulcal pits depth adjusted for age and estimated IQ, while the bottom section shows the mean sulcal pits depth and standard deviation (SD) for each group within that areal

In the left hemisphere, females exhibited deeper sulcal pits than males (Fig. 7, A) in one areal included in the deepest cluster (areal 4), which corresponded to the horizontal ramus of the lateral sulcus and the anterior and posterior parts of the insula according to the Destrieux atlas [44]. The corresponding BA were the 47 and 48. In this areal, 78.42% of the sample presented one sulcal pit and 21.05% presented more than one. Only one male did not present any sulcal pit in this areal.

In the right hemisphere, two areals of the shallowest cluster showed differences regarding depth between sexes. In this case, males exhibited deeper sulcal pits than females (Fig. 7, B and C). Areal 2 comprised the medial orbital sulcus [44] and BA 11 and 25. Areal 35 included the pericallosal sulcus and the middle and posterior parts of the cingulate sulcus [44], and BA 23, 26 and 29. In areal 2, 91.58% of the sample presented one sulcal pit, 4.21% presented more than one and 4.21% did not have any pit (4 males and 4 females). In areal 35, 72.63% of the sample presented one sulcal pit, 9.47% presented more than one and 17.89% did not present any pit (13 males and 21 females).

Linear model results for all the other areals and the percentage of individuals having 1, > 1 or 0 sulcal pits in each areal are presented in Supplementary materials 1, Tables S3 – S4 and Tables S13 – S14, respectively.

## Discussion

Our study aimed to combine the characterization of sex differences in the quantitative patterns of sulcal pits with the analysis of their association with cortical surface area and cortical thickness. Additionally, we investigated the potential role of sulcal pits patterns as markers of early brain formation, using sulcal pits depth as an indicator of their temporal emergence.

First, we found significant differences between males and females in the number of sulcal pits in both hemispheres, with males having a higher number of sulcal pits than females in both hemispheres. This result is not comparable with previous studies analyzing the frequency of sulcal pits since they did not particularly assesses sex differences. The only previous study testing sex effects on sulcal pits [39], based on 1.5T MI data in a sample of healthy 67 males and 67 females, described differences between sexes at some brain regions using structural graph-based morphometry; however, it did not report specifically the direction of such differences in terms of the number of pits. Nevertheless, the fact that sex differences remained marginally significant in the left hemisphere after adjusting for covariates and FDR correction, underscores two main aspects: the potential hemispheric asymmetries in the sulcal pits patterns and the importance of considering these covariates for more accurate analyses.

Concerning the observed age-independent sex differences in sulcal pits frequency at the left hemisphere, our results should be interpreted cautiously due to limited statistical power to detect subtle effects. However, they suggest the interest of new studies to validate whether sex-related differences in sulcal pits frequency are inherently more substantial in the left hemisphere than in the right and therefore pointing towards a hemispheric asymmetry. This is consistent with the study by Takerkart et al. [39], which showed that differences between males and females in the patterns of sulcal pits differed across hemispheres and it also supports previous research suggesting that sulcus-based sex differences are hemisphere-specific [25, 27].

As regards the novelty of testing different covariates and effects not considered by previous studies, our results particularly highlight the role of the ICV and the interaction between sex and age in their anatomical patterns. The significant effect of ICV on the number of sulcal pits aligns with previous studies showing that structural brain differences between sexes may be affected by brain size [50] and that surface area is particularly influenced by it [54]. Furthermore, this study offers an original contribution by examining age-dependent sex differences in sulcal pits patterns. Although sulcal pits have traditionally been considered stable anatomical markers across the lifespan [30, 32], and our results did not reveal a direct effect of age on the number or depth of sulcal pits, we found that aging processes may nonetheless influence the number of sulcal pits in the right hemisphere in a sex-specific manner, as reflected by the significant interaction between age and sex detected in the right hemisphere. This finding aligns with prior evidence suggesting sex-specific trajectories of brain maturation and aging, potentially related to hormonal changes such as those occurring during menopause [55]. Therefore, we hypothesize that the observed interaction effects could be attributed to hormonal changes differentially impacting neurodegenerative processes in females, particularly in midlife and beyond.

Next, in relation to the analysis of sulcal pits correlation with other neuroanatomical features, the observed positive association with surface area in the sex-pooled sample indicates that brains with greater surface area are likely to have a greater number of sulcal pits. This association was not observed when the sample was stratified by sex, likely due to the reduced statistical power resulting from smaller subgroup sizes. Conversely, the negative association of the number of sulcal pits with cortical thickness in the sex-pooled sample is coherent with a study showing how the cortex is thinner in its deepest parts (sulci) compared to the shallowest parts (gyri) [56]. In the left hemisphere, this result appears to be primarily driven by the significant association observed in males, with no corresponding results in females. This disparity may suggest sex-specific divergence in the mechanisms underlying the association between sulcal pits and cortical thickness; however, such interpretations remain speculative and warrant further investigation. In the right hemisphere, although an association was found in the sex-pooled sample, it was less robust than in the left, and it did not reach significance when the sample was stratified by sex. In this sense, similarly to the sulcal pits frequency analyses, the association in the right hemisphere emerges subtler than in the left, equally pointing towards hemisphere asymmetry effects and/or statistical power limitations of our sample.

Second, when we explored sex-specific differences in the number of sulcal pits in each depth cluster, we found no significant result after adjusting for age, estimated IQ and ICV. However, the refined analysis exploring differences in each areal depth unveiled some temporal developmental specificities. Sex differences regarding the depth of sulcal pits emerged in areals within the deep and shallow clusters. While females in the left hemisphere exhibited deeper sulcal pits in an areal corresponding to the deep cluster, males in the right hemisphere presented deeper sulcal pits in areals included in the shallow cluster. More specifically, in the left hemisphere, sex differences in sulcal pits depth were observed in areal 4, which included the insula. This region has been reported to initiate early its development, around 17th – 18th GW [18, 22] and is related to language processing (BA 47), memory encoding and emotion processing (BA 48) [57, 58]. In the right hemisphere, differences between males and females comprised regions on the frontal lobe (the medial orbital sulcus) (areal 2) and the limbic lobe (pericallosal and middle and posterior parts of the cingulate sulcus) (areal 35). The orbital sulcus is developed around 28th GW [22] and the pericallosal and cingulate sulci are developed during the 12th and the 18th GW, respectively [22]. These regions are related to emotional processing and behavior regulation (BA 23–26) and episodic memory and navigation (BA 29) [57]. Based on our analysis, areal 35 was classified within the shallow cluster despite comprising regions developing early in neurodevelopment. It might be due to the variability of sulcal pits occurrence in this areal, as it showed a higher proportion of individuals without any sulcal pit (17.9%) compared to areal 4 (0.5%) and areal 2 (4.2%) (Supplementary Materials 1, Tables S3 – S4).

The regions where we found differences in the depth of sulcal pits between sexes have also been reported in the unique previous study analyzing sex differences in sulcal pits [39]. As well, sex effects have been reported in these regions by voxel-based morphometry approaches [7, 59]. Furthermore, the insula and pericallosal regions have been considered among the more discernible brain features between sexes in a sample of adolescents [60]; and sex differences in the volume of cortical plate regions corresponding to the cingulate and insula, have been reported in fetuses [12]. Conversely, the regions in which we observed sex differences in sulcal pits depth were not among the sulci that contributed most to sex classification in adults based on cortical folding patterns in a previous study [27]. This discrepancy may be explained by the inclusion of additional sulcal features in that study such as length, area, average angle between two neighboring sulci and maximum and minimum depth, which were not examined in our analysis since it focused exclusively on sulcal pits and not the whole sulci morphometry. Although the previous cited study and ours identified sex-specific patterns in different regions, differences consistently emerge from both studies. Taken together, the findings from both studies highlight the potential value of integrating analyses of sulcal pits with broader sulcal morphometric features to better understand sex-specific brain organization.

Despite the role of sulcal pits as early neurodevelopmental markers of brain sex differences reflected by our analysis, a comprehensive understanding of the neurodevelopmental mechanisms underlying these sex-specific patterns requires consideration of additional factors. These differences may be influenced by the combined effects of genetics, hormones, and responses to cellular and external environments, which contribute to sexually differentiated traits through molecular pathways [4]. Interestingly, some of the sex differences in the depth of sulcal pits were detected in brain regions that develop during the peak of testosterone that takes place between 14th – 18th GW, which is suggested to play a role in brain sex differentiation [13]. Furthermore, during mid-gestation, when cortical sulcation occurs, differences in regulatory gene expression in the brain have been reported [61], suggesting sex-biased biological processes involving cell differentiation, energy metabolism, and extracellular matrix organization. Also, during fetal brain development, a sex-differential influence of the placental environment is suggested [62], which may interact with all the afore mentioned biological factors.

In this context, it is worth noting that, in humans, many sex-biased genes are differentially expressed at birth and are primarily localized in the brain, while in other mammals, sex-specific gene expression typically occurs at sexual maturity and is concentrated in other organs [63]. This early and brain-specific differential gene expression in humans may play a role in the development of unique traits associated with brain function and cognition in our species, as well as contribute to their dysregulation, leading to pathological conditions.

Some limitations should be considered in the present study. For instance, the small sample size may be insufficient to capture the full variability in the patterns of sulcal pits. Indeed, our post-hoc power analysis indicated that with 80% power to detect a sex beta of ± 1.69, our study was adequately powered to identify moderate to large effect sizes of sex on the number of sulcal pits, but not small ones. However, we mitigated this limitation by considering those areals with at least one sulcal pit in an 80% of the sample. Also, despite relevant advances in the field [24, 64], the precise knowledge of the timing of sulcal folding remains underexplored, therefore the correlates between sulcal pits emergence and their neurobiological processes need to be cautiously interpreted.

As a final note, while our analyses considered sex as the biological sex assigned at birth, the observed sex-specific differences in sulcal pits patterns may also reflect influences of gender. Prior research has demonstrated that brain development and structure can be shaped by gender-related factors independently of biological sex [6, 65–67]. Therefore, future studies should consider approaches that allow for the differentiation between sex- and gender-related influences on sulcal pits.

### Perspectives and significance

This study enhances our understanding of how sex differences in the patterns of sulcal pits may serve as neurodevelopmental markers, shedding light on the complex interplay between brain structure, surface area, and cortical thickness. By uncovering hemispheric asymmetries in sex-specific sulcal pits patterns and examining their associations with developmental timing and functional regions, our findings emphasize the critical role of sulcal pits in shaping neurodevelopmental trajectories. In particular, our data suggest that deeper and more numerous sulcal pits observed in specific regions may reflect sex-biased neurodevelopmental processes, probably shaped by genetic, hormonal, and environmental factors during early brain development. Notably, the study highlights the necessity of integrating considerations of brain size and asymmetry into future research to disentangle intrinsic sex differences from those driven by overall brain volume. Beyond their developmental relevance, these sex-specific patterns in sulcal pits may offer new insights into sex-biased vulnerabilities to neurodevelopmental disorders, providing a foundation for future studies to investigate their biological underpinnings and clinical implications.

## Conclusions

Our study provides new insights into sex-specific patterns of sulcal pits, revealing significant asymmetries and associations with cortical surface area and thickness. The persistence of sex differences in the left hemisphere after accounting for brain size, estimated IQ and age, highlights asymmetric neuroanatomical variation between the two hemispheres and underscores the importance of considering intrinsic cortical folding patterns in neuroanatomical studies. Additionally, the identified associations between sulcal pits characteristics and brain regions developed at different temporal windows suggest that these features may serve as early markers of sex-biased brain development. While the findings enhance our understanding of the interplay between structural and developmental factors in the brain, they also highlight the need for further research to elucidate the molecular and environmental mechanisms driving these sex-specific patterns and their potential links to neurodevelopmental disorders.

## Electronic supplementary material

Below is the link to the electronic supplementary material.

Supplementary Material 1

Supplementary Material 2

## Data Availability

The data supporting the findings of this study are available from the corresponding authors upon reasonable request.

## References

[1] Díaz-Caneja CM, Alloza C, Gordaliza PM, Fernández-Pena A, De Hoyos L, Santonja J, et al. Sex differences in lifespan trajectories and variability of human sulcal and gyral morphology. Cereb Cortex. 2021;31:5107–20. 34179960 10.1093/cercor/bhab145

[2] Kaczkurkin AN, Raznahan A, Satterthwaite TD. Sex differences in the developing brain: insights from multimodal neuroimaging. Neuropsychopharmacology. Nature Publishing Group; 2019. pp. 71–85. 10.1038/s41386-018-0111-z PMC6235840 29930385

[3] Paus T. Sex differences in the human brain. A developmental perspective. Prog Brain Res Elsevier B V; 2010;186:13–28. 10.1016/B978-0-444-53630-3.00002-6 21094883

[4] Khramtsova EA, Davis LK, Stranger BE. The role of sex in the genomics of human complex traits. Nat Rev Genet Nat Publishing Group. 2019;20:173–90. 10.1038/s41576-018-0083-1 30581192

[5] McCarthy MM, Arnold AP. Reframing sexual differentiation of the brain. Nat Neurosci. 2011;14:677–83. 10.1038/nn.2834 PMC3165173 21613996

[6] Bölte S, Neufeld J, Marschik PB, Williams ZJ, Gallagher L, Lai MC. Sex and gender in neurodevelopmental conditions. Nat Rev Neurol Nat Res; 2023;19:136–59. 10.1038/s41582-023-00774-6 PMC10154737 36747038

[7] Ruigrok ANV, Salimi-Khorshidi G, Lai MC, Baron-Cohen S, Lombardo MV, Tait RJ et al. A meta-analysis of sex differences in human brain structure. Neurosci Biobehav Rev. 2014;39:34–50. 10.1016/j.neubiorev.2013.12.004 PMC3969295 24374381

[8] Kurth F, Gaser C, Luders E. Development of sex differences in the human brain. Cogn Neurosci. 2021;12:155–62. 32902364 10.1080/17588928.2020.1800617 PMC8510853

[9] Peper JS, Burke SM, Wierenga LM. Sex differences and brain development during puberty and adolescence. Handb clin neurol. Elsevier B.V.; 2020. pp. 25–54. 10.1016/B978-0-444-64123-6.00003-5 33008529

[10] Gilmore JH, Knickmeyer RC, Gao W. Imaging structural and functional brain development in early childhood. Nat Rev Neurosci Nat Publishing Group; 2018;19:123–37. 10.1038/nrn.2018.1 PMC5987539 29449712

[11] Wierenga LM, Sexton JA, Laake P, Giedd JN, Tamnes CK. A key characteristic of sex differences in the developing brain: greater variability in brain structure of boys than girls. Cereb Cortex. 2018;28:2741–51. 28981610 10.1093/cercor/bhx154 PMC6041809

[12] Vasung L, Rollins CK, Yun HJ, Velasco-Annis C, Zhang J, Wagstyl K, et al. Quantitative in vivo MRI assessment of structural asymmetries and sexual dimorphism of transient fetal compartments in the human brain. Cereb Cortex. 2020;30:1752–67. 31602456 10.1093/cercor/bhz200 PMC7132947

[13] Pavlinek A, Adhya D, Tsompanidis A, Warrier V, Baron-Cohen S, Allison C, et al. Using organoids to model sex differences in the human brain. Biological Psychiatry Global Open Science. Elsevier Inc.; 2024. 10.1016/j.bpsgos.2024.100343 PMC11292257 39092139

[14] Studholme C, Kroenke CD, Dighe M. Motion corrected MRI differentiates male and female human brain growth trajectories from mid-gestation. Nat Commun. 2020;11. 10.1038/s41467-020-16763-y PMC7297991 32546755

[15] Lohmann G, Von Cramon DY, Colchester ACF. Deep sulcal landmarks provide an organizing framework for human cortical folding. Cereb Cortex. 2008;18:1415–20. 17921455 10.1093/cercor/bhm174

[16] Im K. Cortical sulci in the human fetal brain and development. Factors affecting neurodevelopment: genetics, neurology, behavior, and diet. Elsevier Inc.; 2021. Available from: 10.1016/B978-0-12-817986-4.00031-6.

[17] Namburete AIL, Papież BW, Fernandes M, Wyburd MK, Hesse LS, Moser FA, et al. Normative spatiotemporal fetal brain maturation with satisfactory development at 2 years. Nature. 2023;623:106–14. 37880365 10.1038/s41586-023-06630-3 PMC10620088

[18] Nishikuni K, Ribas GC. Study of fetal and postnatal morphological development of the brain sulci: laboratory investigation. J Neurosurg Pediatr. 2013;11:1–11. 23140215 10.3171/2012.9.PEDS12122

[19] Habas PA, Scott JA, Roosta A, Rajagopalan V, Kim K, Rousseau F, et al. Early folding patterns and asymmetries of the normal human brain detected from in utero MRI. Cereb Cortex. 2012;22:13–25. 21571694 10.1093/cercor/bhr053 PMC3236791

[20] Yun HJ, Lee HJ, Lee JY, Tarui T, Rollins CK, Ortinau CM, et al. Quantification of sulcal emergence timing and its variability in early fetal life: hemispheric asymmetry and sex difference. NeuroImage. 2022;263. 10.1016/j.neuroimage.2022.119629 PMC10011016 36115591

[21] White T, Su S, Schmidt M, Kao CY, Sapiro G. The development of gyrification in childhood and adolescence. Brain Cogn. 2010;72:36–45. 10.1016/j.bandc.2009.10.009 PMC2815169 19942335

[22] Chi JG, Dooling EC, Gilles FH. Gyral development of the human brain. Ann Neurol. 1977;1:86–93. 560818 10.1002/ana.410010109

[23] Long KR, Huttner WB. Formation of gyri and sulci. Patterning and cell type specification in the developing CNS and PNS. Elsevier Inc.; 2020. Available from: 10.1016/B978-0-12-814405-3.00011-4.

[24] Kruggel F. The macro-structural variability of the human neocortex. Neuroimage. 2018;172:620–30. Available from: 10.1016/j.neuroimage.2018.01.074. 10.1016/j.neuroimage.2018.01.074 29410357

[25] Zhao X, Wang Y, Wu X, Liu S. An MRI study of morphology, asymmetry, and sex differences of inferior precentral sulcus. Brain Topogr. 2024. 10.1007/s10548-024-01035-5 PMC11393153 38374489

[26] Wang Y, Xu F, Zhou W, Hou L, Tang Y, Liu S. Morphological and hemispheric and sex differences of the anterior ascending ramus and the horizontal ascending ramus of the lateral sulcus. Brain Struct Funct. 2022;227:1949–61. 35441988 10.1007/s00429-022-02482-1 PMC9232435

[27] Duchesnay E, Cachia A, Roche A, Rivière D, Cointepas Y, Papadopoulos-Orfanos D, et al. Classification based on cortical folding patterns. IEEE Trans Med Imaging. 2007;26:553–65. 17427742 10.1109/TMI.2007.892501

[28] Liu T, Wen W, Zhu W, Trollor J, Reppermund S, Crawford J, et al. The effects of age and sex on cortical sulci in the elderly. NeuroImage. 2010;51:19–27. 20156569 10.1016/j.neuroimage.2010.02.016

[29] Meng Y, Li G, Lin W, Gilmore JH, Shen D. Spatial distribution and longitudinal development of deep cortical sulcal landmarks in infants. Neuroimage. 2014;100:206–18. Available from: 10.1016/j.neuroimage.2014.06.004. 10.1016/j.neuroimage.2014.06.004 PMC4138270 24945660

[30] Le Guen Y, Auzias G, Leroy F, Noulhiane M, Dehaene-Lambertz G, Duchesnay E, et al. Genetic influence on the sulcal pits: on the origin of the first cortical folds. Cereb Cortex. 2018;28:1922–33. 28444225 10.1093/cercor/bhx098 PMC7190941

[31] Auzias G, Brun L, Deruelle C, Coulon O. Deep sulcal landmarks: algorithmic and conceptual improvements in the definition and extraction of sulcal pits. Neuroimage. 2015;111:12–25. Available from: 10.1016/j.neuroimage.2015.02.008. 10.1016/j.neuroimage.2015.02.008 25676916

[32] Im K, Grant PE. Sulcal pits and patterns in developing human brains. Neuroimage. 2019;185:881–90. Available from: 10.1016/j.neuroimage.2018.03.057. 10.1016/j.neuroimage.2018.03.057 PMC6160365 29601953

[33] Hostalet N, Salgado-Pineda P, Martínez-Abadías N, Fatjó-Vilas M. The sulcal pits as neurodevelopmental markers: a systematic review about their potential use in clinical practice. Prog neuropsychopharmacol biol psychiatry. Elsevier Inc.; 2025. pp. 1–12. 10.1016/j.pnpbp.2025.111289 39923914

[34] Brun L, Auzias G, Viellard M, Villeneuve N, Girard N, Poinso F, et al. Localized misfolding within broca’s area as a distinctive feature of autistic disorder. Biol Psychiatry Cogn Neurosci Neuroimaging. 2016;1:160–8. 29560874 10.1016/j.bpsc.2015.11.003

[35] Li X, Wang W, Wang P, Hao C, Li Z. Atypical sulcal pattern in boys with attention-deficit/hyperactivity disorder. Hum Brain Mapp. 2021;42:4362–71. 34057775 10.1002/hbm.25552 PMC8356996

[36] Li X, Jiang YH, Wang W, Liu XX, Li ZY. Brain morphometric abnormalities in boys with attention-deficit/hyperactivity disorder revealed by sulcal pits-based analyses. CNS Neurosci Ther. 2021;27:299–307. 32762149 10.1111/cns.13445 PMC7871795

[37] Lefrere A, Auzias G, Favre P, Kaltenmark I, Houenou J, Piguet C, et al. Global and local cortical folding alterations are associated with neurodevelopmental subtype in bipolar disorders: a sulcal pits analysis. J Affect Disord. 2023. 10.1016/j.jad.2022.12.156 36608853

[38] Im K, Raschle NM, Smith SA, Ellen Grant P, Gaab N. Atypical sulcal pattern in children with developmental dyslexia and at-risk kindergarteners. Cereb Cortex. 2016;26:1138–48. 25576531 10.1093/cercor/bhu305 PMC4757938

[39] Takerkart S, Auzias G, Brun L, Coulon O. Structural graph-based morphometry: a multiscale searchlight framework based on sulcal pits. Med Image Anal. 2017;35:32–45. Available from: 10.1016/j.media.2016.04.011. 10.1016/j.media.2016.04.011 27310172

[40] Gomar JJ, Ortiz-Gil J, McKenna PJ, Salvador R, Sans-Sansa B, Sarró S, et al. Validation of the word accentuation test (TAP) as a means of estimating premorbid IQ in Spanish speakers. Schizophr Res. 2011;128:175–6. Available from: 10.1016/j.schres.2010.11.016. 10.1016/j.schres.2010.11.016 21144711

[41] Nelson HE, Willison J. National adult reading test (NART). Nfer-Nelson Windsor; 1991.

[42] Boucher M, Whitesides S, Evans A. Depth potential function for folding pattern representation, registration and analysis. Med Image Anal. 2009;13:203–14. 18996043 10.1016/j.media.2008.09.001

[43] Im K, Jo HJ, Mangin JF, Evans AC, Kim SI, Lee JM. Spatial distribution of deep sulcal landmarks and hemispherical asymmetry on the cortical surface. Cereb Cortex. 2010;20:602–11. 19561060 10.1093/cercor/bhp127

[44] Destrieux C, Fischl B, Dale A, Halgren E. Automatic parcellation of human cortical gyri and sulci using standard anatomical nomenclature. NeuroImage. 2010;53:1–15. 20547229 10.1016/j.neuroimage.2010.06.010 PMC2937159

[45] Rorden C, Brett M. Stereotaxic display of brain lesions. Behav Neurol. 2000;12:191–200. 11568431 10.1155/2000/421719

[46] Kochunov P, Mangin JF, Coyle T, Lancaster J, Thompson P, Rivière D, et al. Age-related morphology trends of cortical sulci. Hum Brain Mapp. 2005;26:210–20. 16161162 10.1002/hbm.20198 PMC6871665

[47] Nobis L, Manohar SG, Smith SM, Alfaro-Almagro F, Jenkinson M, Mackay CE, et al. Hippocampal volume across age: nomograms derived from over 19,700 people in UK biobank. Neuroimage Clin. 2019;23. 10.1016/j.nicl.2019.101904 PMC6603440 31254939

[48] Lee J, Kim HJ. Normal aging induces changes in the brain and neurodegeneration progress: review of the structural, biochemical, metabolic, cellular, and molecular changes. Front Aging Neurosci. 2022;14. 10.3389/fnagi.2022.931536 PMC9281621 35847660

[49] Im K, Choi YY, Yang JJ, Lee KH, Kim SI, Grant PE, et al. The relationship between the presence of sulcal pits and intelligence in human brains. Neuroimage. 2011;55:1490–6. Available from: 10.1016/j.neuroimage.2010.12.080. 10.1016/j.neuroimage.2010.12.080 21224005

[50] Eliot L, Ahmed A, Khan H, Patel J. Dump the “dimorphism”: comprehensive synthesis of human brain studies reveals few male-female differences beyond size. Neurosci Biobehav Rev. Elsevier Ltd; 2021. pp. 667–97. 10.1016/j.neubiorev.2021.02.026 33621637

[51] Benjamini Y, Hochberg Y. Controlling the false discovery rate: a practical and powerful approach to multiple testing. Journal of the Royal Statistical Society: Series B (Methodological). 1995;57:289–300. Available from: https://rss.onlinelibrary.wiley.com/doi/abs/10.1111/j.2517-6161.1995.tb02031.x.

[52] R Core Team. R: A language and environment for statistical computing. Vienna, Austria: R Foundation for Statistical Computing. 2023. Available from: https://www.R-project.org/.

[53] Schäfer T, Ecker C. fsbrain: an R package for the visualization of structural neuroimaging data. 2020.

[54] Im K, Lee JM, Lyttelton O, Kim SH, Evans AC, Kim SI. Brain size and cortical structure in the adult human brain. Cereb Cortex. 2008;18:2181–91. 18234686 10.1093/cercor/bhm244

[55] Than S, Moran C, Beare R, Vincent AJ, Collyer TA, Wang W, et al. Interactions between age, sex, menopause, and brain structure at midlife: a UK biobank study. J Clin Endocrinol Metab. 2021;106:410–20. 33205159 10.1210/clinem/dgaa847

[56] Holland MA, Budday S, Li G, Shen D, Goriely A, Kuhl E. Folding drives cortical thickness variations. Eur Phys Journal: Special Top. 2020;229:2757–78. 10.1140/epjst/e2020-000001-6 PMC10237175 37275766

[57] Guo Z, Shao C, Zhang Y, Qiu W, Li W, Zhu W, et al. A global multiregional proteomic map of the human cerebral cortex. Genomics Proteom Bioinf. 2022;20:614–32. 10.1016/j.gpb.2021.08.008 PMC9880820 34763096

[58] Strotzer M. One century of brain mapping using brodmann areas. Clin Neuroradiol. 2009;19:179–86. 10.1007/s00062-009-9002-3 19727583

[59] Lotze M, Domin M, Gerlach FH, Gaser C, Lueders E, Schmidt CO, et al. Novel findings from 2,838 adult brains on sex differences in gray matter brain volume. Sci Rep. 2019;9:1671. 10.1038/s41598-018-38239-2 PMC6368548 30737437

[60] Brennan D, Wu T, Fan J. Morphometrical brain markers of sex difference. Cereb Cortex. 2021;31:3641–9. 33774662 10.1093/cercor/bhab037

[61] de Toledo VHC, Feltrin AS, Barbosa AR, Tahira AC, Brentani H. Sex differences in gene regulatory networks during mid-gestational brain development. Front Hum Neurosci. 2022;16. Available from: 10.3389/fnhum.2022.955607https://www.frontiersin.org/articles/. 10.3389/fnhum.2022.955607 PMC9428411 36061507

[62] Rosenfeld CS. The placenta-brain-axis. J Neurosci Res. John Wiley and Sons Inc; 2021. pp. 271–83. 10.1002/jnr.24603 PMC7483131 32108381

[63] Rodríguez-Montes L, Ovchinnikova S, Yuan X, Studer T, Sarropoulos I, Anders S, et al. Sex-biased gene expression across mammalian organ development and evolution. Science. 1979;2023:382. 10.1126/science.adf1046 PMC7615307 37917687

[64] Yun HJ, Vasung L, Tarui T, Rollins CK, Ortinau CM, Grant PE, et al. Temporal patterns of emergence and spatial distribution of sulcal pits during fetal life. Cereb Cortex. 2020;30:4257–68. 32219376 10.1093/cercor/bhaa053 PMC7264701

[65] Luckhoff HK, Smit R, Phahladira L, du Plessis, Emsley R, Asmal L. Sex versus gender associations with brain structure. J Clin Neurosci. 2024;122:103–9. 38493700 10.1016/j.jocn.2024.03.009

[66] Dhamala E, Bassett DS, Yeo BTT, Holmes AJ. Functional brain networks are associated with both sex and gender in children. Sci Adv. 2024;10. 10.1126/sciadv.adn4202 PMC11244548 38996031

[67] Mueller SC, Guillamon A, Zubiaurre-Elorza L, Junque C, Gomez-Gil E, Uribe C, et al. The neuroanatomy of transgender identity: mega-analytic findings from the ENIGMA transgender persons working group. J Sex Med. 2021;18:1122–9. 34030966 10.1016/j.jsxm.2021.03.079

