# Supplementary material for: Sulcal pits as potential markers of early sex-related human brain differences in healthy adults
Source: Biol Sex Differ. 2025 Jul 22;16:55. doi: 10.1186/s13293-025-00733-4 (PMC12281671; doi:10.1186/s13293-025-00733-4)
Supplement: Supplementary file 1 — Supplementary Material 1 [file 13293_2025_733_MOESM1_ESM.docx]

**SUPPLEMENTARY MATERIALS 2**


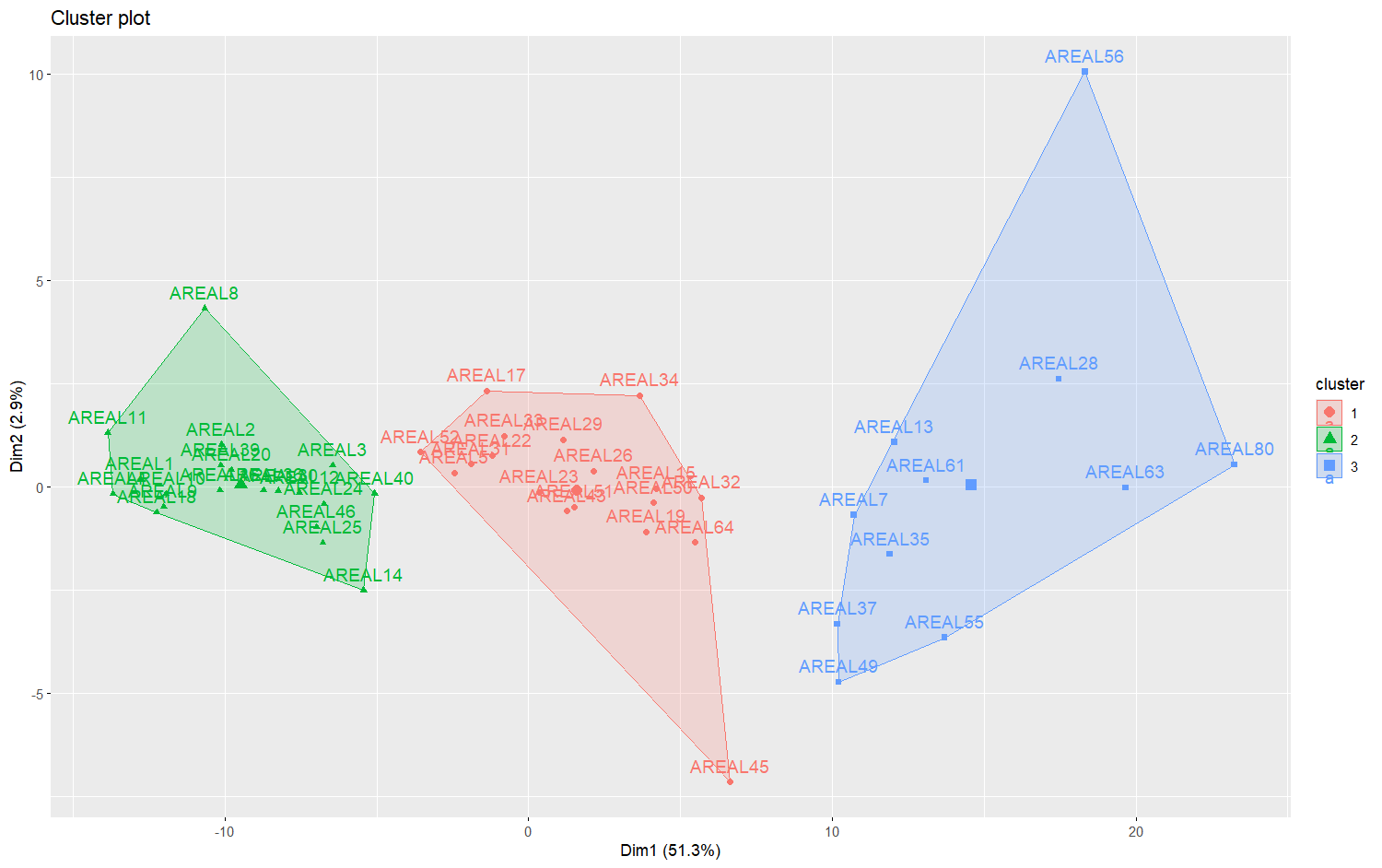


Intermediate

Deep

Shallow

**Figure S1**. Clustering of the areals applying k-means algorithm in the left hemisphere

**Table S1.** Within- and Between cluster sums of squares of k-means performed on areals of the left hemisphere.

| **Left Hemisphere** | | |  |
| --- | --- | --- | --- |
| **Within- cluster sum of squares** | Deep | 123.4756 |  |
|  | Intermediate | 161.9175 |  |
|  | Shallow | 146.6041 |  |
| **Between-cluster sums of squares** | 379.8167 | |  |
|  |  |  |  |


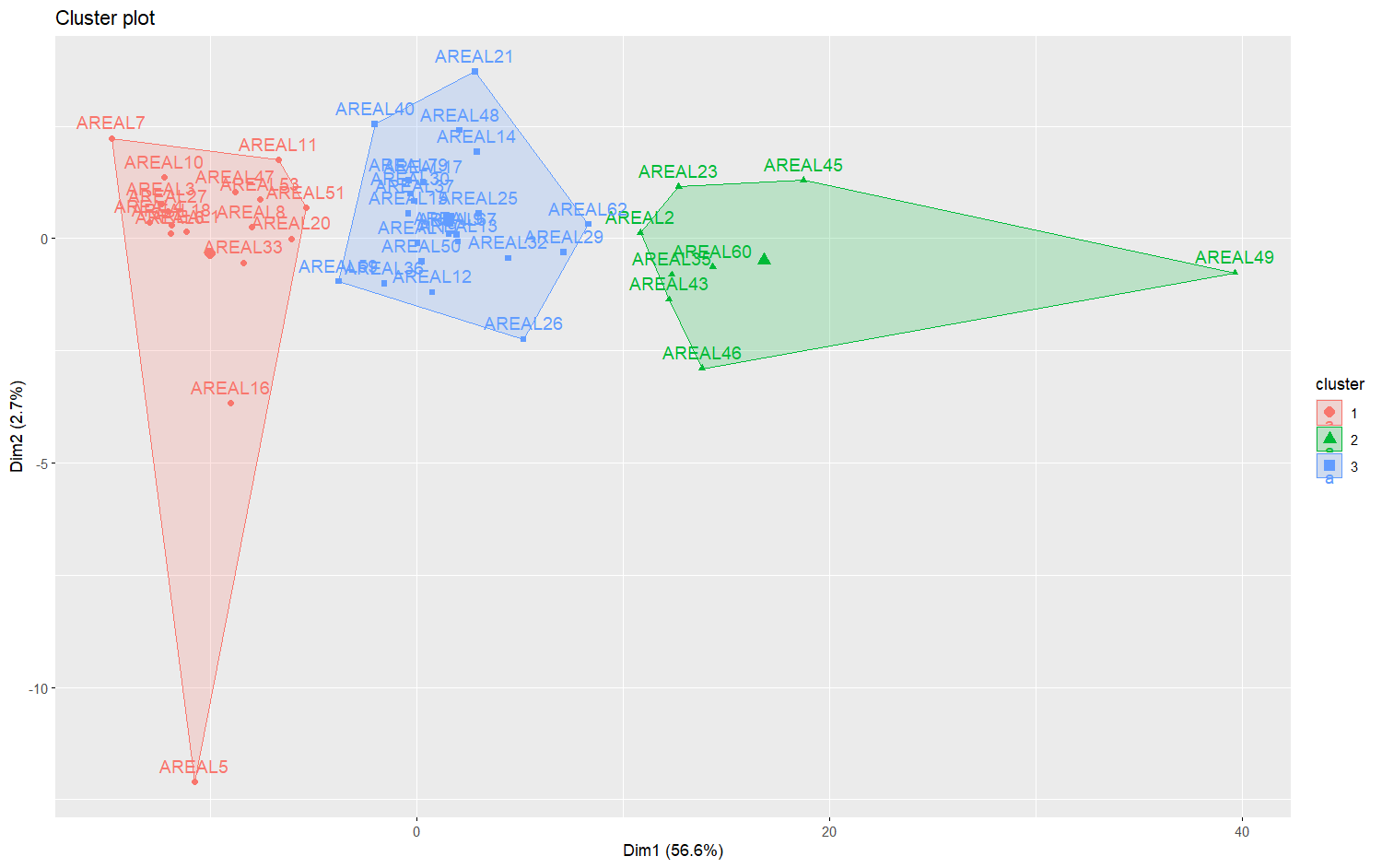


Shallow

Deep

Intermediate

**Figure S2.** Clustering of the areals applying k-means algorithm in the right hemisphere. A second k-means was applied excluding areal 49 that was an outlier in the first dimension (see Table S2 and Figure S2).

**Table S2.** Within- and Between cluster sums of squares of k-means performed on areals of the right hemisphere.

| **Right hemisphere (with outlier areal)** | | |  |
| --- | --- | --- | --- |
| **Within- cluster sum of squares** | Deep | 120.3464 |  |
|  | Intermediate | 189.4840 |  |
|  | Shallow | 130.5500 |  |
| **Between-cluster sums of squares** | 378.5412 | |  |
|  |  |  |  |


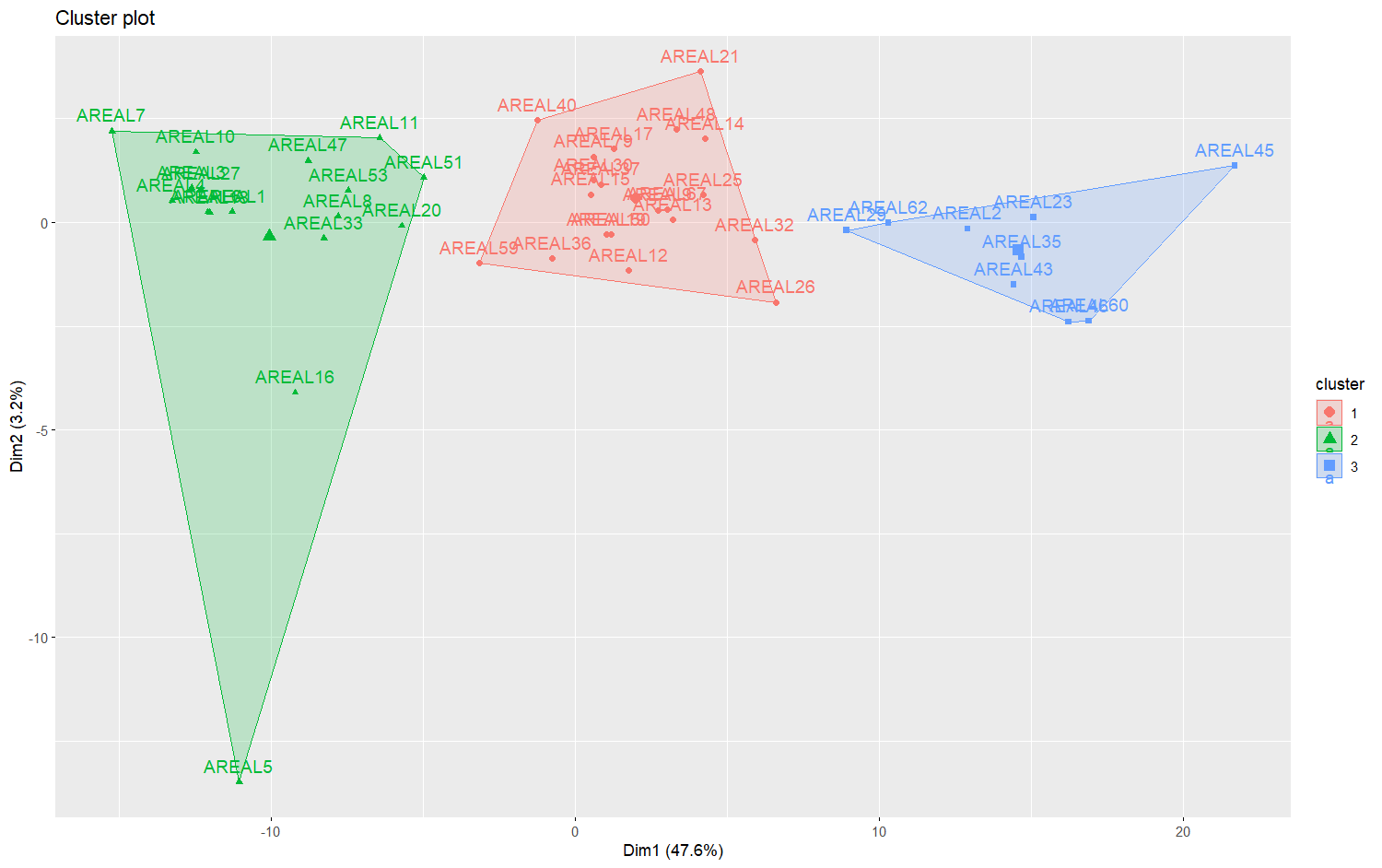


Intermediate

Shallow

Deep

**Figure S3.** Clustering of the areals applying k-means algorithm in the right hemisphere after excluding areal 49.

**Table S3.** Within- and Between cluster sums of squares of k-means performed on areals of the right hemisphere after excluding areal 49

| **Right hemisphere (without areal 49)** | | |  |
| --- | --- | --- | --- |
| **Within- cluster sum of squares** | Deep | 120.3464 |  |
|  | Intermediate | 154.7995 |  |
|  | Shallow | 102.6237 |  |
| **Between-cluster sums of squares** | 292.0195 | |  |
|  |  |  |  |
